# Mutation in the TRKB Cholesterol Recognition Site that blocks Antidepressant Binding does not Influence the Basal or BDNF-Stimulated Activation of TRKB

**DOI:** 10.1007/s10571-023-01438-1

**Published:** 2023-12-17

**Authors:** Caroline Biojone, Cecilia Cannarozzo, Nina Seiffert, Cassiano R. A. F. Diniz, Cecilia A. Brunello, Eero Castrén, Plinio Casarotto

**Affiliations:** 1 Neuroscience Center - HiLife, University of Helsinki, P. O. Box 63, 00014 Helsinki, Finland; 2 Department of Biomedicine, Faculty of Health, Aarhus University, Aarhus, Denmark; 3 Translational Neuropsychiatry Unit (TNU), Department of Clinical Medicine, Aarhus University, Aarhus, Denmark; 4 Center for Neuroscience, University of California, Davis, CA USA

**Keywords:** Tropomyosin-related kinase B receptor, NTRK2, Brain-derived neurotrophic factor, Cholesterol recognition, Alignment consensus

## Abstract

Brain-derived neurotrophic factor (BDNF) acting upon its receptor Neurotrophic tyrosine kinase receptor 2 (NTRK2, TRKB) plays a central role in the development and maintenance of synaptic function and activity- or drug-induced plasticity. TRKB possesses an inverted cholesterol recognition and alignment consensus sequence (CARC), suggesting this receptor can act as a cholesterol sensor. We have recently shown that antidepressant drugs directly bind to the CARC domain of TRKB dimers, and that this binding as well as biochemical and behavioral responses to antidepressants are lost with a mutation in the TRKB CARC motif (Tyr433Phe). However, it is not clear if this mutation can also compromise the receptor function and lead to behavioral alterations. Here, we observed that Tyr433Phe mutation does not alter BDNF binding to TRKB, or BDNF-induced dimerization of TRKB. In this line, primary cultures from embryos of heterozygous Tyr433Phe mutant mice (hTRKB.Tyr433Phe) are responsive to BDNF-induced activation of TRKB, and samples from adult mice do not show any difference on TRKB activation compared to wild-type littermates (TRKB.wt). The behavioral phenotype of hTRKB.Tyr433Phe mice is indistinguishable from the wild-type mice in cued fear conditioning, contextual discrimination task, or the elevated plus maze, whereas mice heterozygous to BDNF null allele show a phenotype in context discrimination task. Taken together, our results indicate that Tyr433Phe mutation in the TRKB CARC motif does not show signs of loss-of-function of BDNF responses, while antidepressant binding to TRKB and responses to antidepressants are lost in Tyr433Phe mutants, making them an interesting mouse model for antidepressant research.

## Introduction

Brain-derived neurotrophic factor (BDNF) is a critical regulator of neuronal connectivity and plasticity, and these effects are mediated by binding of BDNF to neurotrophic tyrosine kinase receptor 2 (NTRK2, TRKB) (Bothwell 2016). Neuronal plasticity is compromised in several brain disorders and promotion of plasticity would be highly useful in the prevention and treatment of as well as in recovery and rehabilitation after several neurological and psychiatric disorders (Castrén and Antila 2017). Indeed, there is a widespread consensus that BDNF-TRKB-mediated plasticity is a critical mediator of the effects of antidepressant drugs and recent evidence indicates that TRKB is the direct site of action of antidepressant binding (Casarotto et al. 2021).

Cholesterol is another important regulator of synaptic maturation and plasticity (Mauch et al. 2001; Martín et al. 2014). Dietary cholesterol does not pass into the brain and the nervous system is dependent on locally produced cholesterol (Martín et al. 2014). The majority of neuronal cholesterol is produced by astrocytes that provide neurons with cholesterol through an ApoE-mediated transport (Pfrieger 2003; Martín et al. 2014). Cholesterol is an integral component of plasma membrane and cholesterol concentration within different plasma membrane compartments varies from low levels to up to 50 mol% in synaptic membranes (Ikonen 2008; Tulodziecka et al. 2016). Cholesterol concentrations in synaptic membranes increase during postnatal development, along with synaptic maturation (Tulodziecka et al. 2016) and in the absence of cholesterol, synapses fail to mature and synaptic signaling is compromised (Mauch et al. 2001).

Several lines of evidence suggest that TRKB is a major target through which the effects of cholesterol on neuronal plasticity are mediated. BDNF signaling increases the cholesterol production in neurons (Suzuki et al. 2013; Zonta and Minichiello 2013) and TRKB signaling is regulated by cholesterol (Suzuki et al. 2004; Pereira and Chao 2007). We have recently shown that TRKB, but not other TRK-family members, possesses an inverted cholesterol recognition domain (CARC) within the transmembrane region (Cannarozzo et al. 2021; Casarotto et al. 2021).

Cholesterol apparently influences TRKB function through at least two mechanisms. First, through direct interaction with TRKB through the CARC domain, as a tyrosine to phenylalanine point mutation in the position 433 (TRKB.Tyr433Phe), which is considered a critical component of the CARC domain (Fantini et al. 2019), blocks the facilitatory effects of cholesterol on TRKB signaling (Casarotto et al. 2021). Second, and our data suggest that this is the main effect, cholesterol regulates the configuration of TRKB transmembrane domains (TMD) through its effects on membrane thickness: membranes rich in cholesterol, such as synaptic membranes, are thicker, which influences the positioning of the TRKB TMDs. TRKB TMD possesses a ^439^AXXXG^443^ dimerization motif, through which the TMDs of TRKB dimers crisscross each other within the plasma membrane, analogous to the EGF receptor (Arkhipov et al. 2013; Endres et al. 2013; Sinclair et al. 2018). In moderate cholesterol concentrations, TRKB assumes a crossed configuration that is competent for efficient BDNF signaling. In thick membranes, however, the crossed configuration is disrupted and TRKB is not stable, which is consistent with the previous observations that TRKB is excluded from thick lipid raft membranes (Suzuki et al. 2004; Pereira and Chao 2007). Indeed, our previous data suggest that cholesterol has a bell-shaped effect on TRKB signaling: moderate increase in plasma membrane cholesterol promotes TRB signaling, while very low and high concentrations inhibit it (Casarotto et al. 2021). This effect appears to be mostly mediated by a decreased TRKB localization at the cell surface in high cholesterol concentrations (Cannarozzo et al. 2021), which is consistent with the exclusion of TRKB from synaptic membranes (Suzuki et al. 2004; Pereira and Chao 2007).

We recently discovered that antidepressant drugs, including the typical serotonin selective reuptake inhibitors (SSRI) and tricyclic antidepressants (TCA) as well as the rapid-acting antidepressant ketamine, directly bind to the TRKB CARC domain, thereby stabilizing the signaling-competent TRKB structure in synaptic membranes and allosterically increasing BDNF signaling through TRKB (Casarotto et al. 2021). Indeed, transgenic mice heterozygous for a Tyr433Phe point mutation in the CARC domain fail to show any plasticity-promoting and antidepressant-like behavioral responses of antidepressant administration (Casarotto et al. 2021). It is unclear, however, whether the TRKB.Tyr433Phe mutation has any effects on basic expression or localization of TRKB, or on the ability of BDNF to activate and signal through TRKB, and whether such effects might contribute to the inability of antidepressants to influence the mutant TRKB.

We have here investigated the effects of the TRKB.Tyr433Phe mutation on baseline activity of TRKB and on the effects of BDNF through the mutated receptor in vitro and in vivo. As our previous studies have shown that the effects of antidepressant drugs are lost in heterozygous mice with one Tyr433Phe mutant allele, we have focused on the analysis of the heterozygous mutants. We found that heterozygous TRKB.Tyr433Phe mutation (hTRKB.Tyr433Phe) has no effects on the baseline signaling of TRKB, which is consistent with our earlier observations that Tyr433Phe mutation does not influence baseline TRKB localization or signaling (Casarotto et al. 2021). Furthermore, we found no effects of TRKB.Tyr433Phe signaling on BDNF binding or plasticity-related behavioral responses that are sensitive to BDNF. These data suggest that the hTRKB.Tyr433Phe mutation has no effect on the normal function of TRKB, but it is not sensitive to the effects of typical antidepressant drugs or ketamine.

## Material and Methods

### Animals

Male and female mice (C57BL/6JRccHsd background, Envigo-Harlan Labs, Netherlands) heterozygous to TRKB.Tyr433Phe or TRKB.wt littermates were used when weighing about 25–30 g. They were bred and genotyped as described previously (Casarotto et al. 2021). For comparison, male adult mice of C57BL/6J-000664 background (from Jackson Laboratories), carrying a deletion in one of the copies of *Bdnf* gene (BDNF.het) or BDNF.wt littermates were used (Karpova et al. 2011).

The mice (maintained in the Laboratory Animal Center of the University of Helsinki) were 16–18 weeks old at the beginning of the experiments, group-housed (3–6 per cage—type ll: 552 cm^2^ floor area, Tecniplast, Italy) under a 12 h light/dark cycle, with free access to water and food. All protocols were approved by the ethics committee for animal experimentation of Southern Finland (ESAVI/38503/2019).

### Cell Culture and Sample Collection

Cultures of cortical cells from mouse embryos were prepared as previously described in detail (Sahu et al. 2019). Briefly, suspended cortical cells from each embryo were seeded in poly-L-lysine (Sigma-Aldrich, Cat# P4707)-coated 24-well plates (Thermo-Fisher Scientific—USA, cat#144530) at 250,000 cells/well. The cells were maintained in Neurobasal medium (Thermo-Fisher, Cat# 21103049), supplemented with B27 (Thermo-Fisher, Cat# 17504001) and left undisturbed, except for medium change (1/3 twice per week). During the culture preparation samples from each embryo were collected for genotyping as described previously (Casarotto et al. 2021).

Mouse neuroblastoma cell line N2A were cultured as described previously (Casarotto et al. 2021). Briefly, the cells were cultivated in DMEM (Lonza, cat# BE12-614Q), supplemented with 10% v/v inactivated fetal bovine serum (Sigma-Aldrich, Cat# F9665), 1% v/v l-glutamine (Thermo-Fisher, Cat# 25030081), and penicillin/streptomycin (Lonza, cat# DE17-603E), at 37 °C in 5% CO_2_.

### Direct Binding Assay—DBA

The cell-free assays (binding assays) were performed in white 96-well plates (PerkinElmer, OptiPlate 96F-HB, Cat# 6005500) as described before (Casarotto et al. 2021). The plates were precoated with anti-GFP antibodies (Abcam, #ab290, 1:1000) in carbonate buffer (pH 9.8), ON at 4 °C. Following blocking with 3% bovine serum albumin (BSA, Sigma-Aldrich, Cat# A9418) in PBS buffer (2 h at RT), 120 μg of total protein from each sample (of lysates from HEK293T cells transfected to overexpress GFP-TRKB.wt, GFP-TRKB.Tyr433Phe or GFP-TRKB/TRKA.TM) were added and incubated overnight at 4 °C under agitation. The plates were then washed 3 × with PBS buffer, and various concentrations of biotinylated BDNF (Alomone Labs, cat#B-250, 0.1–100 pM) were added for 1 h at RT. The luminescence was determined via HRP-conjugated streptavidin (Thermo-Fisher, cat#21126, 1:10,000, 1 h, RT) activity reaction with ECL (Thermo Scientific Cat# 32106) by a plate reader. The luminescence signal from blank wells (containing all the reagents but the sample lysates, substituted by the blocking buffer) was used as background. The specific signal was then calculated by subtracting the values of blank wells from the values of the samples with matched concentration of the biotinylated ligand.

### Enzyme-Linked Immunosorbent Assay—ELISA

The levels of phosphorylated TRKB in primary cortical cultures of mouse embryos were determined by ELISA as previously described (Fred et al. 2019; Casarotto et al. 2021). Briefly, the cells were homogenized in cold NP lysis buffer (20 mM Tris–HCl, 150 mM NaCl, 50 mM NaF, 1% Nonidet-40, 10% glycerol) supplemented with 2 mM Na_3_VO_4_ and cOmplete protease inhibitor mix (Roche, Cat# 4,693,116,001), centrifuged (4 °C, 15,000 g, 10 min) and stored at − 80 °C. For the ELISA assay, goat anti-TRKB antibody (R&D System, #AF1494) was diluted 1:500 in carbonate buffer pH 9.7 (25 mM sodium bicarbonate, 25 Mm sodium carbonate) and coated in flat-bottom white plates (PerkinElmer, OptiPlate 96F-HB, Cat# 6005500) overnight at 4 °C under agitation. Unbound antibody was then removed from the plate, and 5% BSA in PBST was incubated for 2 h at RT to block non-specific binding sites. The samples were thawed on ice, transferred to the ELISA plate, and incubated overnight under agitation at 4 °C. The plates were washed 3 × with PBST and incubated overnight at 4 °C under agitation with rabbit anti-phosphorylated TRKB (Cell Signaling, either #4168 against pY816 or #4619 against pY515) diluted 1:1000 in 5% BSA/PBST. After a washing step (3 × PBST), the plates were incubated with 1:5000 anti-rabbit HRP-conjugated tertiary antibody (BioRad, Cat#170-5046) for 2 h at RT. Plates were washed again in PBST, ECL (Thermo Scientific Cat# 32106) was added, and the luminescence was detected by Varioskan Flash plate reader (Thermo Scientific). Unspecific signal (from wells where sample was omitted) was assessed in each plate and subtracted from samples signal. Final data were expressed as percentage of the control (vehicle treated group).

### Western Blotting—WB

The levels of pTRKB and total TRKB in the prefrontal cortex of experimentally naive adult male mice were determined by WB as previously described (Rantamäki et al. 2007). Samples were homogenized as mentioned above and denatured in a 2X Laemmli buffer (BioRad, Cat# 1610737) for 5 min at 95 ℃. SDS-PAGE was carried out by resolving the samples by electrophoresis in NuPAGE 4–12% Bis–Tris Protein polyacrylamide gels (Cat# NP0323BOX, Invitrogen). After the electrophoresis, the samples were transferred to a PVDF membrane and incubated in primary antibody diluted 1:1000 in 3% BSA/TBST overnight at 4 ℃. The membrane was subsequently washed and incubated in HRP-conjugated secondary antibody against the appropriate host (1:10,000, BioRad) for 1 h of RT. The bands were visualized using Pierce™ ECL Plus western blotting substrate (#32132 Thermo-Fisher Scientific). Primary antibodies used were rabbit anti-phosphorylated TRKB against Y515, Y706, or Y816 (Cell Signaling #4619, #4621, and #4168, respectively); goat anti-TRKB (R&D System, #AF1494) or anti-β-actin mouse monoclonal antibody (Sigma-Aldrich, #A1978). Considering that HEK293T cells virtually do not express TRKB, all the antibodies against TRKB were validated by comparing non-transfected cells with TRKB-transfected cells (data not shown). To ensure the quality of the signal, the membranes were stripped only once.

### Protein-Fragment Complementation Assay—PCA

Protein-fragment complementation assay (PCA) measures the reconstitution of enzymatic activity of a humanized *Gaussia princeps* luciferase (GLuc) following a direct interaction of the proteins of interest (Kim et al. 2009; Merezhko et al. 2020; Casarotto et al. 2021). Two complementary fragments of the luciferase reporter protein were fused to the intracellular C terminus of TRKB or mutant TRKB.Tyr433Phe to produce the wild-type TRKB PCA pair (GLuc1C-TRKB.wt/GLuc2C-TRKB.wt) and the heterozygous TRKB.Tyr433Phe PCA pair (GLuc1C-TRKB.wt/GLuc2C- TRKB.Tyr433Phe). The GLuc tag was linked via a GS linker that allows the physiological dynamics of TRKB without interference from the presence of the tag. When two TRKB molecules carrying the complementary GLuc fragments dimerize, the reporter refolds in its active conformation thereby producing bioluminescence in the presence of its substrate native coelenterazine. Neuro2A cells, in 10% (v/v) poly-l-Lysine (Sigma-Aldrich, Cat# P4707) coated white 96 wells (PerkinElmer, OptiPlate 96F-HB, Cat# 6005500; 10,000 cells/well) were transfected with the above-mentioned PCA pair constructs. Cells were treated 48 h post-transfection with BDNF (10 ng/ml/10 min), and luminescence measured as a direct indication of TRKB homodimerization with a plate reader (Varioskan Flash, Thermo Scientific, average of 5 measurements, 0.1 s each) immediately after the injection of the coelenterazine substrate (Nanolight Technology, Cat# 3031).

### Sholl Analysis of Neurite Branching in Cortical Cultures

Cortical neurons were incubated with BDNF (Peprotech Cat#450-02) 20 ηg/ml or veh for 15 min once a day for 3 days (7, 8, and 9 DIV in the afternoon). At 9 DIV (in the morning), the neurons were incubated with MgCl_2_ 10 µM for 1 h to prevent excitotoxicity and then transfected with mCherry construct, using lipofectamine 2000 (Thermo-Fisher, Cat# 11668019) following the manufacturer’s instructions. The culture medium was replaced by a fresh Neurobasal medium (Thermo-Fisher, Cat# 21103049) supplemented only with l-glu (Thermo-Fisher, Cat# 25030081), B27 (Thermo-Fisher, Cat# 17504001), and penicillin/streptomycin (Lonza, cat# DE17-603E) 90 min after the transfection. 24 h after the last BDNF treatment, the coverslips were fixed with PFA 4% for 20 min, blocked, and stained with chicken polyclonal anti-MAP2 1:5,000 (#ab5392, Abcam) and Hoescht (Thermo-Fisher, Cat# H1399) 1:10,000 for 10 min. Coverslips were washed in PBST (3x) and miliQ water once, then mounted in DAKO fluorescence mounting media (Cat# S3023, Dako North America, Inc.). Imaging was acquired in Zeiss LSM700 confocal microscope, 25 × oil objective at 1024 × 1024 pixel resolution, using 647 ηm (for MAP2) and 568 ηm (mCherry) channels. At least 10 Z-stack steps were acquired. Confocal pictures were analyzed in ImageJ (Fiji) software, by compiling the 568 ηm z-stacks, setting the threshold automatically (Shanbhag threshold, available in the software), setting the center of the soma manually, and then counting the branching intersections automatically with built-in Sholl analysis tool (Ferreira et al. 2014). Sholl circle interval was set to 5 µm and the intersections were counted up to 100 µm of distance from the soma.

### Behavioral Analysis

All behavioral trials in the present study were conducted in experimentally naive animals.

#### Cued Fear Conditioning—CuedFC

Male TRKB.Tyr433Phe mice and their TRKB.wt littermates were submitted to the fear conditioning protocol to tone followed by two extinction trials, as described previously (Karpova et al. 2011). Briefly, mice were first cue/shock conditioned by co-terminating 5 tone conditioned stimuli (CS of 80 dB, 1 Hz, 30 s) with an unconditioned stimulus footshock (US of 0.6 mA, 1 s) in an inter-trial interval of 20–120 s. Over the next 2 days, the freezing-conditioned response was extinguished by subjecting mice to 12 of these same tones (inter-trial interval of 20–60 s) each day. Fear conditioning and extinction were performed in different contexts, respectively, context A of transparent Plexiglas walls with metal grids on the floor and context B of non-transparent black Plexiglas walls with a flat floor. For each behavioral procedure, mice remained undisturbed in the experimental room for 1 h for acclimatization, and mice were allowed to explore the environment for 2 min before the presentation of the first tone. The chamber was cleaned with 70% ethanol solution before each animal change. Freezing levels are presented as the sum of all freezing time spent on every tone trial within each extinction session and were determined by the software (TSE, Bad Homburg, Germany).

#### Context Discrimination Task—CDT

Male TRKB.Tyr433Phe mice and their TRKB.wt littermates were submitted to a context discrimination task as described previously (Michels et al. 2018; Laukkanen et al. 2021). Briefly, the animals were acclimated to the experimental room for 1 h, following 5-min habituation to the experimental chamber, the animals received 3 scrambled shocks (0.6 mA/2 s, intervals 30 s^−1^ min) in context A (conditioning, transparent walls, LxWxH: 23 × 23 × 35 cm, with metal grid bottom) followed by a 2-min period without any shocks. On the following day, the animals were exposed to the unfamiliar context B (black walls with the same dimensions, and black sleek bottom) in a 5-min session. On day 3, the animals returned to the familiar context A for 5 min. The time spent in freezing (s) was determined by the software (TSE, Germany) in the full 5 min for each context.

#### Elevated Plus Maze—EPM

Male and female TRKB.Tyr433Phe mice and their TRKB.wt littermates were submitted to the elevated plus maze as described previously (Laukkanen et al. 2021). Briefly, the animals were acclimated to the experimental room for 1 h, and submitted (5 min session) to an acrylic-built elevated plus maze. The apparatus was composed of two open arms (30 × 5 cm, a 1 cm rim to prevent falls) and two enclosed arms (same dimensions with a 25 cm wall). The arms were connected by a central platform (5 × 5 cm) and the apparatus was elevated 40 cm above the floor. The percentage of time and entries in the open arms were determined by the software (Ethovision XT 13, Noldus, Netherlands).

### Statistical Analysis

ELISA, Sholl, and PCA experiments, as well as CuedFC, and CDT were analyzed by two-way ANOVA followed by Fisher’s LSD or Tukey’s (for Sholl) when appropriate. WB and EPM data were analyzed by Student’s t test. We have not run any statistical method to predetermine sample sizes as they were estimated according to similar experiments previously described in the literature (Casarotto et al. 2021). For all data we computed the relative probability of sampling from a Gaussian (normal) vs. a lognormal distribution. The Shapiro–Wilk test was then run to confirm the normal distribution of our data. We assumed homoscedasticity of all groups. The distribution of samples or animals between the groups was randomized and the experimenter was always blind to the treatment and mice genotype. All data used in the present study, including the prism files and an excel file with tables describing the normality tests, are stored on figshare under a CC-BY license (10.6084/m9.figshare.24164421).

## Results

### Tyr433Phe Does not Interfere with BDNF Activation of TRKB In Vitro

**Fig. 1:**
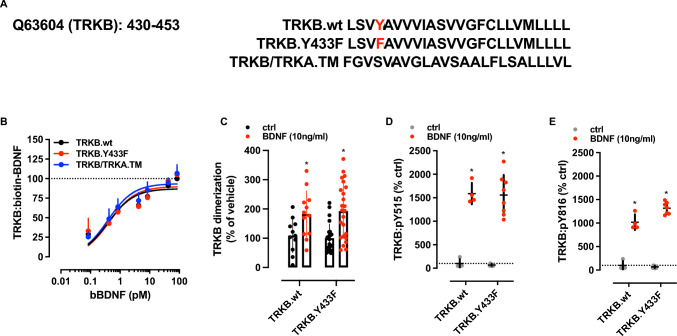
*Y433F does not interfere with BDNF activation of TRKB.* **A** The sequence of TRKB transmembrane (TM) domain from rat (UniProt: Q63604, residues 430–453) was mutated at Y433 residue (Y433F) or the entire motif was substituted by the rat sequence of TRKA TM (P35739, residues 419–442). **B** The interaction between biotinylated BDNF (bBDNF) and TRKB is not affected by the TRKB.Y433F mutation or in TRKB/TRKA.TM constructs (two-way ANOVA). **C** TRKB.Y433F does not prevent the BDNF-induced dimerization of TRKB. The constructs used allow the formation of TRKB.wt homodimer or TRKB.wt/Y433F heterodimer (two-way ANOVA). **D**, **E** Analysis of TRKB phosphorylation shows that cortical cultures from TRKB.Y433F heterozygous mouse embryos respond to BDNF similarly to the TRKB.wt littermates, regardless of the tyrosine residue tested **D** Y515,** E** Y816, two-way ANOVA). *P < 0.05 from ctrl group [see main parameters and exact *P* values in the results section]. Data presented as mean ± SD

First, binding assays were performed to verify whether the Y433 residue is in any way relevant for BDNF to bind to TRKB. Thus, Y (tyrosine) 433 residue from rat TRKB was mutated to F (phenylalanine), or the whole TRKB TM domain was replaced with the rat TRKA sequence (TRKB/TRKA.TM), as indicated in Fig. 1A. As seen in Fig. 1B, the Tyr433Phe mutation or the TRKA transmembrane domain in TRKB do not alter the binding of bBDNF to TRKB. The dissociation constant (Kd ± SEM, pM, *n* = 6/group) observed were TRKB.wt = 0.3904 ± 0.0877; TRKB.Tyr433Phe = 0.4375 ± 0.1069; TRKB/TRKA.TM = 0.3874 ± 0.0747.

Next, the protein fragment complementation assay was used to evaluate any influence of Tyr433Phe upon BDNF action on TRKB dimerization. Expectedly, the Tyr433Phe mutation in TRKB did not prevent the BDNF-induced dimerization of this receptor as BDNF induced an increase in the dimerization of TRKB.wt, while no alteration on BDNF-induced dimerization of the heterodimer TRKB.wt/Tyr433Phe was observed by the two-way ANOVA [interaction: *F*(1,67) = 0.2658, *P* = 0.6079; genotype: *F*(1,67) = 0.0006266, *P* = 0.9801; BDNF: *F*(1,67) = 20.13, *P* < 0.0001, *N* = 11–13/group]; Fig. 1C.

Further, phospho-TRKB levels were used as a proxy for TRKB activation. The effect of BDNF on TRKB phosphorylation (pTRKB) was retained in primary cortical cultures from TRKB.Y433F mutant mice (*N* = 4–8/group). No effect of Tyr433Phe on BDNF-induced pTRKB levels was observed by two-way ANOVA on pTRKB at Y515 [interaction: *F*(1,20) = 0.002, *P* = 0.9962; genotype: *F*(1,20) = 0.07156, *P* = 0.7918; BDNF: *F*(1,20) = 160.1, *P* < 0.0001, Fig. 1D] or at Y816 [interaction: *F*(1,19) = 12.98, P = 0.0019; genotype: *F*(1,19) = 8.389, *P* = 0.0093; BDNF: *F*(1,19) = 557.7, *P* < 0.0001]; Fig. 1E.

### Tyr433Phe Does not Interfere with the TRKB-Dependent Action of BDNF on Neuritogenesis

**Fig. 2:**
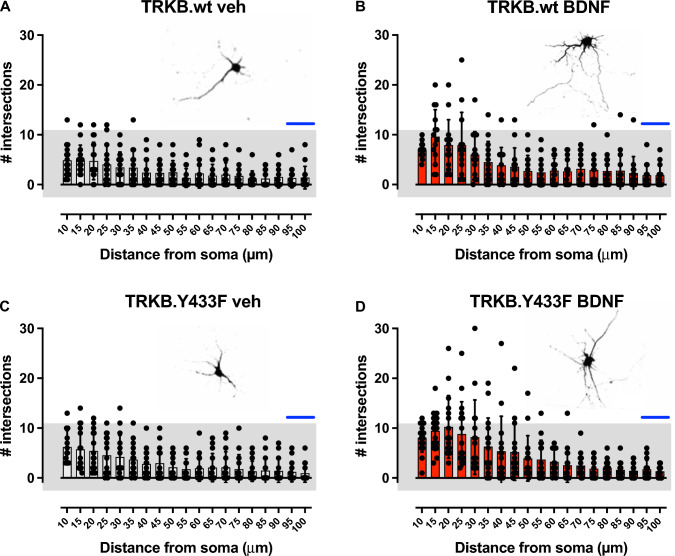
*Y433F does not interfere with the TRKB-dependent action of BDNF on Neuritogenesis*. Sholl analysis indicates that there was no basal difference in relation to neuritogenesis between cortical neurons cultured from TRKB.wt (upper panels, **A** and **B**) and TRKB.Y433F heterozygous (bottom panels, **C** and **D**) mice. Additionally, the neurons respond similarly to BDNF treatment (right panels in red, **B** and **D**), regardless of the genotype (two-way ANOVA). Data presented as mean ± SD, blue scale bar: 50 μm [see main parameters and exact *P* values in the results section]

Sholl analysis technique was chosen to follow-up with our intention to observe whether the effects of BDNF on neuroplasticity would be somehow affected by the Tyr433Phe mutation. Actually, BDNF effectively induced neuritogenesis in cortical cultures regardless of the genotypes investigated (Fig. 2). Two-way ANOVA identified effect of the distance from soma [*F*(18,1064) = 20.51, *P* < 0.0001] and the groups [TRKB.wt veh, TRKB.Tyr433Phe veh, TRKB.wt BDNF, TRKB.Tyr433Phe BDNF; *F*(3,1064) = 23.79, *P* < 0.0001], but no interaction between those factors [*F*(54, 1064) = 1.021, *P* = 0.4353]. Tukey’s Multiple Comparison Test pointed to a significant effect of BDNF in neurons cultured from both WT (veh vs BDNF: *P* < 0.0001) and TRKB.Tyr433Phe mutant mice (veh vs BDNF: *P* < 0.0001). No difference was found when comparing TRKB.wt veh *versus* TRKB.Tyr433Phe veh (*P* = 0.7921), nor TRKB.wt BDNF *versus* TRKB.Tyr433Phe BDNF (*P* = 0.3636).

### Tyr433Phe Does not Interfere with Endogenous BDNF-Dependent TRKB Activation in the Prefrontal Cortex of Adult Mice

**Fig. 3:**
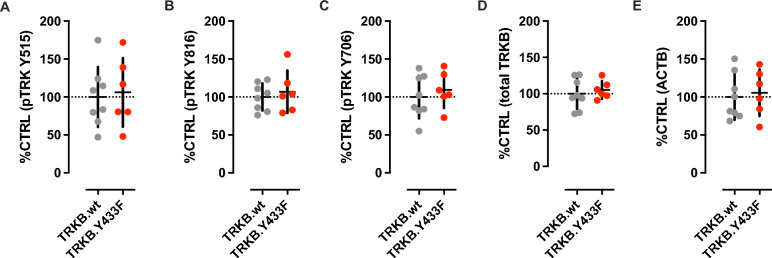
*Y433F does not interfere with endogenous BDNF-dependent TRKB activation in the prefrontal cortex of adult mice.* The basal levels of phosphorylated TRKB are not different in the prefrontal cortex of TRKB.wt and TRKB.Y433F mice (**A**: Y515, **B**: Y816, **C**: Y706, **D**: total TRKB, **E**: beta-actin, ACTB, Student’s *t* test) [see main parameters and exact *P* values in the results section]. Data presented as mean ± SD

Adult mice were now used to confirm whether a more complex brain structure than cultured cortical neurons would keep TRKB.Tyr433Phe immune to the effects of endogenous BDNF on pTRKB levels. Indeed, in adult TRKB.Tyr433Phe mice the pTRKB levels measured in the prefrontal cortex were not affected by the genotype [Student’s *t* test; pTRKB Y515: *t*(12) = 0.2665, *P* = 0.7944; Y816: *t*(12) = 0.5650, *P* = 0.5825; Y706/7: *t*(12) = 0.6503, *P* = 0.5277; total TRKB: *t*(12) = 0.5396, *P* = 0.5993; beta-actin, ACTB: *t*(12) = 0.3391, *P* = 0.7404; *N* = 6/group], as seen in Fig. 3A–E.

### No Behavioral Phenotype is Found Altered in TRKB.Tyr433Phe Mice

**Fig. 4:**
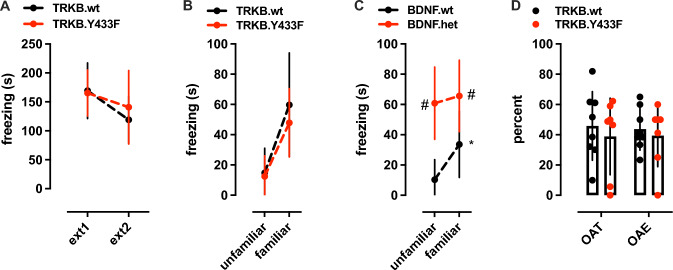
*No behavioral phenotype is found altered in TRKB.Y433F mice*. **A** TRKB.Y433F mice exhibit a similar extinction profile of cued fear conditioning response to TRKB.wt mice (two-way ANOVA with repeated measures). This strain also did not show any deficits in fear extinction to context [see (Casarotto et al. 2021)]. **B** TRKB.Y433F mice are able to discriminate between familiar and unfamiliar contexts previously associated with footshocks (two-way ANOVA with repeated measures). **C** For comparison, BDNF.het mice show impaired response to the context discrimination task (two-way ANOVA with repeated measures). **D** TRKB.Y433F mice do not show any changes in the anxiety-related parameters of elevated plus maze (Student’s *t* test). *P < 0.05 from ext1 or unfamiliar context; #*P* < 0.05 from BDNF.wt group in the same trial [see main parameters and exact *P* values in the results section]. Data presented as mean ± SD

Finally, a battery of tests was performed with TRKB.Tyr433Phe mice to assess whether these mice display any behavioral phenotype, despite the Tyr433Phe mutation having no apparent basal phenotype with respect to the effects of BDNF. First, cued fear conditioning paradigm indicates that no genotype effect of Tyr433Phe mutation was observed on the extinction of conditioned fear reaction [interaction: *F*(1,19) = 0.8641, *P *= 0.3643; trial: *F*(1,19) = 7.367, *P* = 0.0138; genotype: *F*(1,19) = 0.2931, *P* = 0.5945, *N* = 10,11], as seen in Fig. 4A.

No genotype effect of Tyr433Phe mutation was observed in the ability to discriminate between conditioned and unconditioned contexts [two-way ANOVA with repeated measures; interaction: *F*(1,15) = 0.6472; *P* = 0.4337; trial: *F*(1,15) = 45.87; *P* < 0.0001; genotype: *F*(1,15) = 0.5711, *P* = 0.4615; *N* = 8,9], Fig. 4B. For comparison, BDNF.het mice have compromised performance in this test [two-way ANOVA with repeated measures; interaction *F*(1,10) = 6.745, *P* = 0.0266; trial: *F*(1,10) = 15.64, *P* = 0.0027; genotype: *F*(1,10) = 12.57, *P* = 0.0053; *N* = 6/group], Fig. 4C, as observed previously (Laukkanen et al. 2021), as well as in the extinction of conditioned fear (Karpova et al. 2011).

As seen in Fig. 4D, no effect of the genotype was also observed in the performance on the EPM [student’s t test; percent time in the open arm, %OAT: *t*(13) = 0.5657, *P* = 0.5813; percent entries in the open arm, %OAE: *t*(13) = 0.4779, *P* = 0.6407, *N* = 8 (wt), 7 (Tyr433Phe)].

## Discussion

Our findings demonstrate that hTRKB.Tyr433Phe mutation has no effects on the baseline or BDNF-induced TRKB signaling, or on TRKB-mediated behavior. This confirms our previous findings that the hTRKB.Tyr433Phe mutation has no effect on basal TRKB signaling, TRKB plasma membrane localization, and on basal behavior of hTRKB.Tyr433Phe mutant mice (Casarotto et al. 2021). In sharp contrast, essentially all the effects of antidepressants and ketamine were lost in these mice (Casarotto et al. 2021). These data suggest that this heterozygous mutation selectively inhibits the effects of drugs that directly bind to the TRKB CARC domain without having any effects on basic or BDNF-induced TRKB signaling, which makes this mouse an excellent experimental model for the antidepressant research.

Binding of BDNF to TRKB.Tyr433Phe was indistinguishable from that of the wild-type TRKB. Moreover, substitution of the entire TMD of TRKB by that of TRKA also had no effects of BDNF binding. TRKA does not have the ^439^AXXXG^443^ dimerization motif and its TMD is also in other ways divergent from that of TRKB (Nikoletopoulou et al. 2010), which suggests that the TMD configuration does not influence BDNF binding. BDNF binds to the second Ig-domain of TRKB (Schneider and Schweiger 1991) and this binding is apparently independent of the nature of the TMD.

We have used split luciferase-based protein complementation assay to investigate the effects of the TRKB.Tyr433Phe mutation on TRKB dimerization (Remy and Michnick 2006). Cells co-expressed the TRKB.Tyr433Phe mutant and the wild-type TRKB, each tagged with a complementary half the luciferase reporter. Therefore, dimerization that is revealed in this assay can only occur in a heterozygous configuration, between a mutant and a wild-type TRKB. We found that BDNF-induced dimerization is intact with this assay. We previously found that TRKB dimerization was reduced, although not completely prevented, in a homozygous configuration where two TRKB.Tyr433Phe mutants carrying complementary fragments of the luciferase were overexpressed (Casarotto et al. 2021).

Cultured cortical neurons derived from hTRKB.Tyr433Phe mice showed no differences in the baseline or BDNF-induced TRKB autophosphorylation in response to BDNF administration. Our previous studies showed that when the TRKB.Tyr433Phe construct is overexpressed in cultured cells, the response to BDNF stimulation is compromised (Casarotto et al. 2021). Specifically, autophosphorylation of the Y816, a recognition site for PLCγ, is reduced when TRKB.Tyr433Phe is overexpressed, while autophosphorylation of the Y515, the shc recognition site, is not compromised. Here, we have found that both of these responses are normal in neurons derived from the hTRKB.Tyr433Phe mice and the baseline phosphorylation of both these sites is also normal in brain tissue from hTRKB.Tyr433Phe mice. Taken together, these findings suggest that for the baseline activity and BDNF responses, TRKB.Tyr433Phe mutation acts as a recessive mutation, but for responses to antidepressant drugs, it acts as a dominant, apparent already when a single allele of TRKB is mutated. This conclusion was also supported by the behavioral findings with the hTRKB.Tyr433Phe mice. While these mice exhibit a dramatically reduced responsiveness to antidepressants (Casarotto et al. 2021), they showed normal behavior in tests that are known to be sensitive to BDNF signaling. In contrast, mice heterozygous for BDNF null allele show clear behavioral deficits in the context discrimination test, confirming that this response requires intact BDNF signaling.

An exome sequencing study of 197 unrelated patients with developmental and epileptic encephalopathy identified four patients with a similar phenotype and a Tyr433Cys (Tyr434Cys in human nomenclature) de novo point mutation in the TRKB gene, in the very same tyrosine residue that has been mutated to phenylalanine in our studies (Hamdan et al. 2017). While more research is needed to elucidate the exact mechanism of action of this Tyr433Cys mutation, it is possible that the developmental and epileptic encephalopathy is produced by a constitutive active TRKB through a disulfide bridge between the mutant cysteine residues. Being a de novo mutation and therefore heterozygous, a pair of mutant alleles that can create a S–S bridge is only out of four possible TRKB dimers, nevertheless, only a minority of TRKB dimers when constitutively active may produce a dramatic phenotype. Since mutations of a tyrosine to both cysteine and phenylalanine require a point mutation in only a single nucleotide (A > G and A > U, respectively) and several Y433 mutations have been detected, it is likely that also the Tyr433Phe mutation (or Tyr434Phe in human TRB) has occurred, however, we have not found that mutation reported in repositories. Our current data indicate that a human TRKB.Tyr434Phe mutation would likely be recessive and not produce any overt phenotype as heterozygous, which may explain why it has not been detected so far. Nevertheless, these findings highlight the importance of the tyrosine-433 (434 in case of humans) for TRKB signaling.

It is worth noting that our in vitro cell culture approach used the benefits of technical replicates. Although this approach is very useful for characterizing a specific protocol-based mechanism, it ignores biological diversity, which may be relevant to any inference about a broader population-based effect.

Taken together, we have found that a hTRKB.Tyr433Phe mutation does not interfere with the baseline TRKB function or with BDNF signaling and it does not produce any phenotype in behavioral tests that are known to be sensitive to BDNF signaling. However, plasticity-promoting and antidepressant-like responses to antidepressant drugs that bind to TRKB at the site including the tyrosine-433 are severely compromised in the hTRKB.Tyr433Phe mice, indicating that while this mutation is recessive for BDNF responses, it is dominant for antidepressant binding. The hTRKB.Tyr433Phe mouse could therefore be of substantial interest in the process of screening for novel potential antidepressants or other drugs that promote plasticity by binding to the TRKB transmembrane domain.

## Data Availability

All data used in the present study is stored in FigShare under a CC-BY license (10.6084/m9.figshare.20655042).
